# Knowledge, Attitude, Practice, and Barriers Regarding Prediabetes Among Adults in Saudi Arabia

**DOI:** 10.7759/cureus.67699

**Published:** 2024-08-24

**Authors:** Rawan Alsalman, Abdulrahman J Alsharari, Anwar N AlmohammedAli, Azzam Alzahrani, Basel S Alghamdy, Nawaf Alzibali, Raghad Alshamrani, Rahaf Z Al-Beladi, Waleed A Alasmari, Waseem Jadoh, Ahmed Jaradat

**Affiliations:** 1 Medicine, Arabian Gulf University, Manama, BHR; 2 Family and Community Medicine, Arabian Gulf University, Manama, BHR

**Keywords:** prediabetes screening, attitude, barriers, practice, knowledge, prediabetes in saudi arabia

## Abstract

Background

Prediabetes refers to a clinical condition in which blood glucose levels are elevated but do not meet the threshold for diabetes. Prediabetes is now thought to be reversible; lifestyle changes and other interventions can be successfully implemented during the prediabetes phase to avoid the development of type 2 diabetes. This study aims to improve health outcomes among Saudi community members who are at risk of developing prediabetes by assessing their knowledge, attitudes, practices, and barriers using a validated prediabetes questionnaire.

Methods

This study employed a cross-sectional design across various regions of Saudi Arabia. It included patients who were all non-diabetic Saudi adults over 18 years old and visited outpatient clinics. Structured questionnaires, which included participants’ demographic information, knowledge, attitudes, practices, and barriers related to prediabetes, were employed. The collected data were analyzed using Statistical Product and Service Solutions (SPSS; IBM SPSS Statistics for Windows, Armonk, NY) software program.

Results

Data from 641 patients were collected in this survey. The respondents were predominantly female (330, 51.5%), with the mean ± SD of age being 36.3 ± 12.3. The participant knowledge levels of prediabetes were found to be good (399, 62.2%), moderate (193, 30.1%), and poor (49, 7.6%). The knowledge scores were significantly associated with age (P = 0.027), educational level (P < 0.001), education in the medical field (P = 0.019), and monthly family income (P = 0.009). The overall attitude of the participants toward prediabetes was generally positive (468, 73%). The practices related to diet and lifestyle among the participants were generally poor (538, 84%). Some participants did not take blood sugar tests because they were not available (121, 18.9%), lacked time (179, 27.9%), and were afraid of learning the test results (130, 20.3%).

Conclusion

The study found that, despite possessing an adequate level of knowledge and positive attitudes, Saudi patients poorly practice prevention methods for prediabetes. It highlights the need for targeted interventions to improve prediabetes awareness, promote healthier lifestyles, and address screening barriers. Prioritizing evidence-based strategies that cater to diverse demographic needs can prevent the progression of type 2 diabetes and enhance public health. The findings emphasize the importance of health education in Saudi Arabia and suggest that future research should focus on overcoming barriers, such as management complexity, diagnosis apprehension, and time constraints for check-ups.

## Introduction

Prediabetes refers to a clinical condition in which blood glucose levels are elevated but do not meet the diabetes threshold. According to the American Diabetes Association (ADA), people without diabetes are classified as having prediabetes if they have a glycated hemoglobin (HbA1c) value between 5.7% and 6.4%, impaired fasting glucose (IFG) between 100 and 125 mg/dL, or impaired glucose tolerance (IGT) between 140 and 199 mg/dL [1].

Studies have suggested that people with prediabetes are at a high risk of developing complications of diabetes, including cardiovascular diseases, nephropathy, and neuropathy [2]. Moreover, in Saudi Arabia, 25.5% of the population aged ≥30 years is living with prediabetes, putting more than three million people at risk for diabetes mellitus (DM). It is concerning that, annually, 5-10% of individuals with prediabetes progress to type 2 DM (T2DM); in addition, 40.3% of diabetic patients are unaware that they have the disease [3].

Prediabetes is now thought to be reversible; lifestyle changes and other interventions can be successfully implemented during the prediabetes phase to avoid the development of T2DM [4]. A few studies conducted in Saudi Arabia have determined the knowledge, attitudes, practices, and barriers of prediabetes among the Saudi community. This study conducts surveys to assess the same variables among adults in Saudi Arabia. Therefore, it aims to improve health outcomes among members of the Saudi community who are at risk of developing prediabetes.

## Materials and methods

Study area and population 

This is a cross-sectional study conducted for two months from 01/08/2023 to 01/10/2023 in Saudi Arabia. The study population included 641 non-diabetic Saudi adults 18 years of age or older who visited outpatient clinics at seven hospitals in Saudi Arabia. These were King Fahad Hospital in Al Hofuf, National Guard Hospital in Riyadh, East Jeddah General Hospital in Jeddah, Mahyal Assir General Hospital in Mahyal, Dammam Medical Complex in Dammam, King Fahad Central Hospital in Abu Arish, and Prince Metaab bin Abdelaziz Hospital in Sakakah.

Sample size and sampling technique

The sample size (n) was calculated according to the following formula: n = z^2^ × p × (1 − p) / e^2^.

Here, z is 1.96 for a confidence level (α) of 95%, p is 0.23 for proportion, and e is 0.05 for the margin of error. (Based on a previous study [5], about 23% of the population was knowledgeable about prediabetes.) Using these parameters, the study determined that a minimum sample size of 273 participants was required and would help in effectively investigating the research objectives with adequate statistical power and precision. In the present study, 641 participants were recruited. The sampling technique involved convenience sampling, in which participants were selected based on their availability and willingness to participate in the study.

Inclusion criteria

The patients who were included in the study were all non-diabetic Saudi adults 18 years of age or older who visited outpatient clinics.

Exclusion criteria

Non-Saudi patients, diabetic patients, pregnant women, children under 18 years old, and emergency cases were excluded from the study.

Data collection tools

The study included a self-administered questionnaire distributed by the authors to non-diabetic Saudi citizens in Saudi Arabia. However, the questionnaire was in Arabic and had closed-ended questions. Moreover, the questionnaire comprised five sections. The first section gathered sociodemographic data, including gender, age, nationality, occupation, education level, education in the medical field, and monthly family income. The second section evaluated patients' knowledge about prediabetes with 11 questions with true or false answers. Following this, the third section gauged attitudes toward prediabetes via a 5-point Likert scale (a rating system that allows respondents to express a range of opinions with five response options), with options beginning from "strongly agree" to "strongly disagree" under seven questions. The fourth section was designed to explore the participants' prevention practices for prediabetes with seven questions. Finally, the fifth section consisted of six questions concerning the barriers they faced to prediabetes screening on the 5-point Likert scale, beginning from "strongly agree" to "strongly disagree."

Further, a knowledge scoring system was developed mathematically. For each knowledge statement, 1 point was given for a "correct" answer and zero for an "incorrect" answer. The knowledge score ranged from 0 to 11, with a minimum score of zero and a maximum score of eleven. We divided this range into three equal intervals. The level of knowledge was categorized as poor (three or fewer correct answers), moderate (between four and seven correct answers), and good (eight or more correct answers). In the third and fifth sections, which used Likert scales, the questionnaire combined the option "strongly agree" with "agree" and the option "strongly disagree" with "disagree" in the results section.

Study variables

Independent variables: These variables comprise demographic data, including age, gender, educational level, employment status, and family income.

Dependent variables: These variables include knowledge, attitudes, practices, and barriers.

Ethical considerations

Approval was obtained from the ethical review committee of Arabian Gulf University to conduct this study (E6-PI-4-23/Group 6). Moreover, the administrations of the seven hospitals also received approval. The participants' privacy was ensured, and the data were kept confidential. All participants were informed about the aim of the study, and their informed consent was obtained. They were informed that the data would be used only for research and statistical analysis.

Statistical analysis

Data analysis was conducted using Statistical Product and Service Solutions (SPSS; IBM SPSS Statistics for Windows, Armonk, NY) software program, and descriptive statistics were employed to summarize the data. Categorical variables were described using counts and percentages to present a clear understanding of the distribution within the sample. The analysis enabled a comprehensive examination of demographic characteristics, knowledge, attitudes, and barriers regarding prediabetes.

## Results

Demographic characteristics

For this survey, 641 questionnaires were collected. As shown in Table 1, the respondents were predominantly female (330, 51.5%), and only 311 respondents (48.5%) were male. Among the 641 respondents, 256 (39.9%) were over 40 years old, 211 (32.9%) were 25-40 years old, and only 174 (27.1%) were 18-25 years old. The majority of the respondents (388, 60.5%) were holders of bachelor's degrees. Following this, 128 respondents (20%) had high school-level education and below, and 73 respondents (11.4%) were diploma holders. The postgraduates held the last position (52, 8.1%). In terms of occupation, 251 participants (39.2%) were government officials, 136 (21.2%) were students, 100 (15.6%) were private-sector employees, 73 (11.4%) were homemakers, and 39 (6.1%) and 42 (6.6%) were unemployed and retired, respectively. Further, 512 of the respondents (79.9%) had no education in the medical field. The majority of the respondents (210, 32.8%) earned more than 12,000 SAR as monthly income; 192 (30%) earned more than 3,000 SAR; 89 (13.9%) earned 3,000-5,999 SAR; and 78 (12.2%) and 72 (11.2%) earned 9,000-12,000 SAR and 6,000-8,999 SAR as monthly income, respectively.

**Table 1:** Sociodemographic characteristics of the participants ^1^The mean ± SD of age was 36.3 ± 12.3.

Question	Option	n (%)
Sex	Male	311 (48.5)
	Female	330 (51.5)
Age^1^	18-25 years	174 (27)
	25–40 years	211 (33)
	>40 years	256 (40)
Educational level	High school and below	128 (20)
	Diploma	73 (11.4)
	Bachelor's degree	388 (60.5)
	Master's degree	52 (8.1)
Occupation	Student	136 (21.2)
	Government official	251 (39.2)
	Private-sector employee	100 (15.6)
	Unemployed	39 (6.1)
	Retired	42 (6.6)
	Homemaker	73 (11.4)
Education in the medical field	Yes	129 (20.1)
	No	512 (79.9)
Monthly family income	<3,000 SAR	192 (30)
	3,000–5,999 SAR	89 (13.9)
	6,000–8,999 SAR	72 (11.2)
	9,000–12,000 SAR	78 (12.2)
	>12,000 SAR	210 (32.8)

Knowledge of prediabetes

Table 2 illustrates the prediabetes knowledge of the participants in different regions of Saudi Arabia. In addition, 427 participants (66.6%) responded correctly regarding the blood sugar level, verifying that a patient is in the prediabetic stage. Only 250 participants (39%) had sufficient knowledge about the progression of prediabetes to diabetes. More than half of the participants (390, 60.8%) answered correctly regarding the chances of developing prediabetes if their parents were diagnosed with diabetes. In fact, the best method for detecting prediabetes is a blood test, and the estimated number of participants who entered optimal answers was 524 (81.7%). Further, 474 participants (73.9%) indicated that prediabetes is a reversible condition, and 258 (40.2%) had prior knowledge of sugar levels in the prediabetic stage. Additionally, 580 participants (90.5%) were aware of the medical recommendations made for prediabetes patients to adopt a healthy diet and exercise. In addition, most of the participants (581, 90.6%) indicated that prediabetes patients are recommended to have a high-fiber diet and exercise regularly. On being asked a question regarding the right exercise duration, only 408 participants (63.7%) responded correctly. On being asked regarding weight loss and its relationship with improvement in prediabetes among obese people, 459 participants (71.6%) responded with the correct answer. In response to the question regarding screening individuals over 45 years of age who have no risk factors, 527 out of 641 participants (82.2%) provided the correct answer.

**Table 2:** Prediabetes knowledge of the participants

Knowledge statement	Right answer	Number of correct answers, (%)
The prediabetic condition occurs when blood glucose levels are higher than normal but are not high enough to be diagnosed as diabetes.	True	427 (66.6)
The prediabetic condition cannot lead to type 2 diabetes mellitus (T2DM).	False	250 (39)
There is no chance an individual could develop prediabetes if both their parents have type 2 diabetes.	False	390 (60.8)
A blood test is the best method of detecting prediabetes.	True	524 (81.7)
The fasting blood glucose level (after an overnight fast of 10 h) in prediabetes is 100–125 mg/dL.	True	258 (40.2)
Prediabetes patients are recommended to have a high-fiber diet and exercise regularly.	True	581 (90.6)
Prediabetes is an irreversible condition that cannot be treated.	False	474 (73.9)
The recommendations for prediabetes are diet control and exercise.	True	580 (90.5)
Prediabetes patients should exercise once a month for at least one hour.	False	408 (63.7)
Weight loss does not improve prediabetes in obese patients.	False	459 (71.6)
An adult who is over 45 years of age should be screened for prediabetes, even if they do not have any risk factors.	True	527 (82.2)

Attitudes toward prediabetes

Table 3 presents the attitudes of the 641 participants toward prediabetes using a 5-point Likert scale. In the survey, 469 respondents (73%) agreed that prediabetic patients should keep their blood sugar levels within normal ranges, 369 (57%) disagreed with the statement that managing blood sugar is difficult if the patient is prediabetic, and 394 (61.5%) disagreed with the statement that controlling blood sugar levels for prediabetes would prevent the development of T2DM. The majority of the participants (566, 88.3%) agreed that people with prediabetes should be educated about DM. While 487 participants (76%) claimed that prediabetes is ignored by society, 570 (88.9%) believed that managing prediabetes requires the help of the patient's family. Finally, 422 participants (65.8%) stated that they could live a normal life with prediabetes.

**Table 3:** Participants' attitudes regarding prediabetes * Correct attitudes given by respondents.

Statement suggesting their attitude	Disagree n (%)	Neutral n (%)	Agree n (%)
Patients with prediabetes should keep their blood sugar level close to normal.	64 (10)	108 (16.8)	469 (73.2)*
The control of blood sugar is difficult with prediabetes.	369 (57.6)*	148 (23.1)	124 (19.3)
Even if the blood sugar level is controlled in prediabetes, T2DM will happen anyway.	394 (61.5)*	174 (27.1)	73 (11.4)
People with prediabetes should be taught about diabetes mellitus (DM).	35 (5.5)	40 (6.2)	566 (88.3)*
The condition of prediabetes is ignored by society.	61 (9.5)	93 (14.5)	487 (76)*
Family support is important in dealing with prediabetes.	22 (3.4)	49 (7.6)	570 (88.9)*
I can have a normal life even after developing prediabetes.	90 (14)	129 (20.1)	422 (65.8)*

Prediabetes prevention practices 

**Figure 1:**
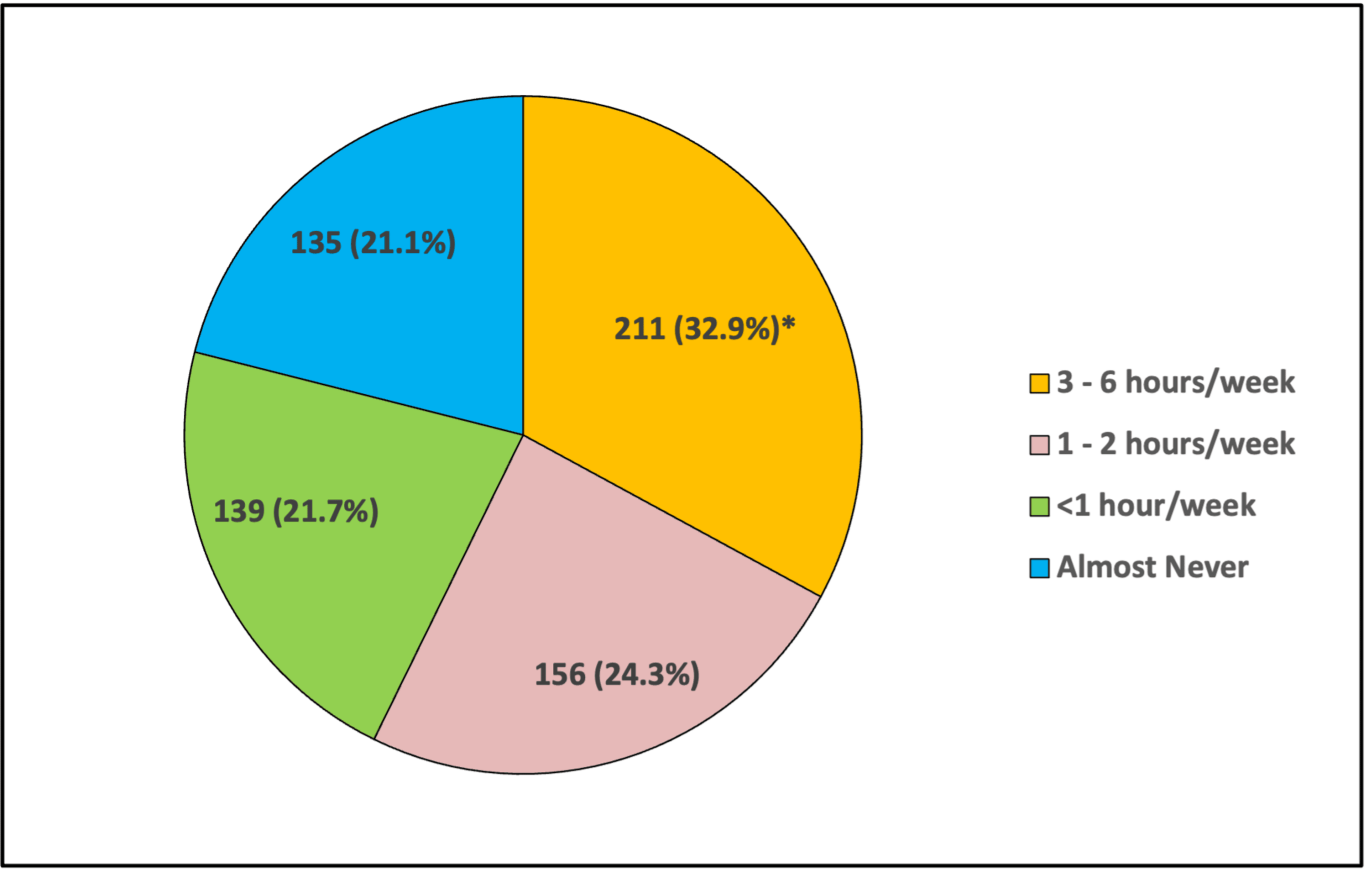
Hours of exercise, such as cycling, walking, and yoga (performed per week) * Correct answers given by respondents.

Figure 1 shows the distribution of responses to the question "How many hours per week do you engage in exercises such as cycling, walking, and yoga?" The findings suggest that 21% (135) of the respondents exercised for three to six hours per week, while 33% (211) exercised for one to two hours per week. However, notably, 22% (139) reported exercising for less than one hour per week, and 24% (156) reported almost never exercising.

**Figure 2:**
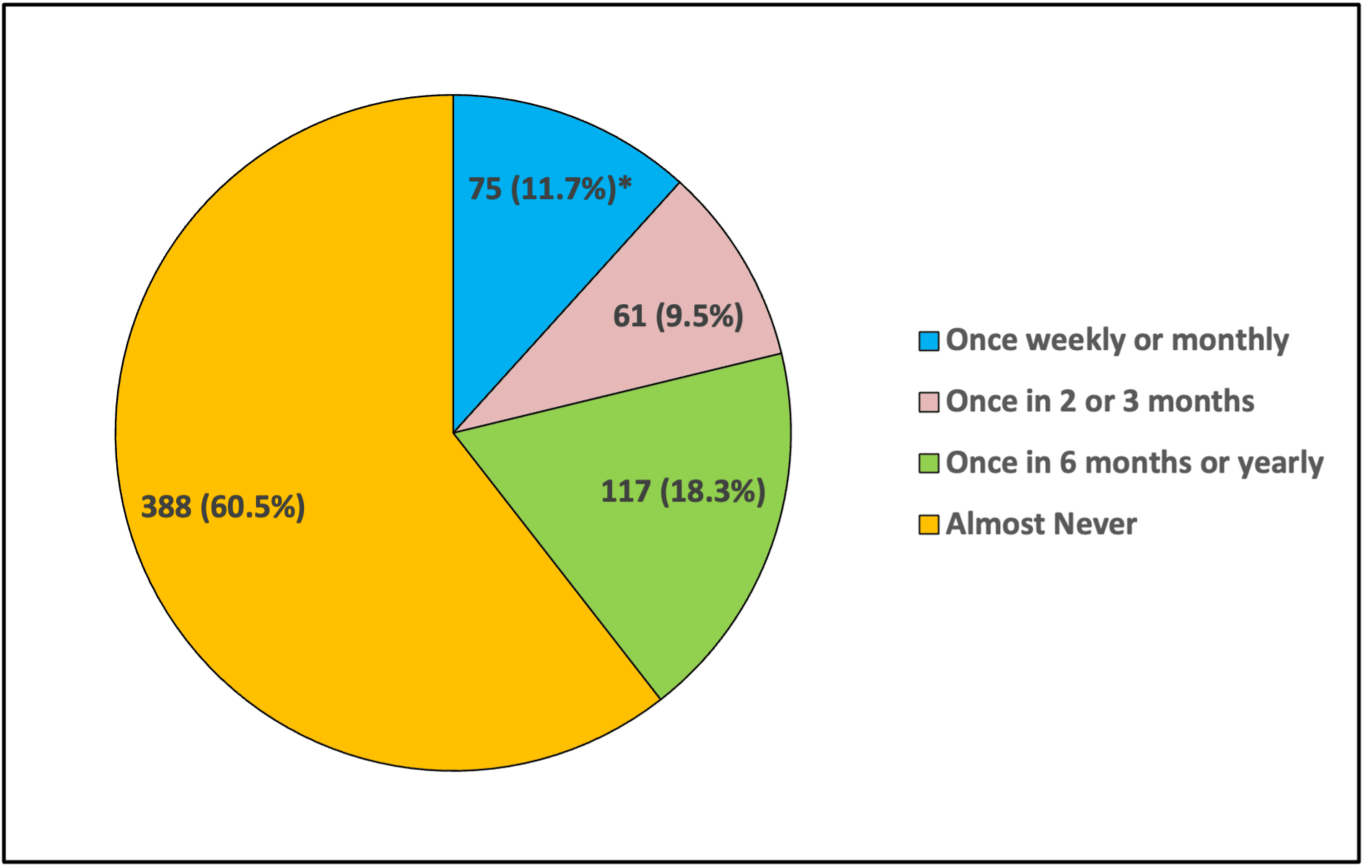
Blood sugar check at home or at a laboratory * Correct answers given by respondents.

Figure 2 shows the relative proportion of how often the participants checked blood sugar at home or at a laboratory for prediabetes screening. These data are listed as follows: once weekly or monthly, once in two or three months, once in six months or yearly, and almost never. Many participants (388, 66.5%) did not check their blood sugar, which is "almost never," whereas 18.3% (117) checked their blood sugar once every six months or a year. Further, 11.7% (75) of the participants checked their blood sugar once a week or month, while less than 9.5% (61) checked their blood sugar once every two or three months.

Table 4 presents information on the prediabetes prevention practices followed by the participants, revealing a diverse range of habits. A small percentage of the respondents (123, 19.2%) claimed they "almost never" consume sugar-sweetened beverages. Only 14.5% (93) of the participants made a conscious effort to adopt a fiber-rich diet, opting for such food over normal meals five or more times a week. Furthermore, the sleep patterns of the surveyed individuals were also found to vary. The majority (232, 36.2%) reported having inadequate sleep one to two times per week, while a smaller segment (101, 15.8%) reported that they "almost never" experienced insufficient sleep. The majority (236, 36.8%) skipped meals three to four times a week, while 68 participants (10.6%) claimed that they "almost never" skipped meals. The consumption of high-fat foods also varied, with 237 (37%) consuming them one to two times per week and 53 (8.3%) claiming that they "almost never" consumed them.

**Table 4:** Prediabetes prevention practices followed by the participants * Correct answers given by respondents.

Question regarding practices	≥5 times/week n (%)	3–4 times/week n (%)	1–2 times/week n (%)	Almost never n (%)
How often do you consume sugary beverages (such as soda, carbonated beverages, and non-carbonated fruit drinks)?	132 (20.6)	140 (21.8)	246 (38.4)	123 (19.2)*
How frequently do you substitute fiber-rich foods (such as oats, whole grains, fruits, and vegetable salads) over normal meals?	93 (14.5)*	196 (30.6)	254 (39.6)	98 (15.3)
How often do you sleep less than six hours per night?	110 (17.2)	198 (30.9)	232 (36.2)	101 (15.8)*
How often do you skip meals?	148 (23.1)	236 (36.8)	189 (29.5)	68 (10.6)*
How often do you consume high-fat foods (such as fried snacks, meat, fast food, and chocolates)?	131 (20.4)	220 (34.3)	237 (37)	53 (8.3)*

Barriers to prediabetes screening

Table 5 shows the prevalence of screening barriers faced by the 641 participants, 397 of whom (61.9%) did not take a blood sugar test because it was not available and 121 of whom (18.9%) did take it. Further, 340 participants (53%) disagreed with the statement that the lack of time is an issue in taking a blood sugar test, and 179 (27.9%) believed that the lack of time is a barrier. The majority (529, 82.50%) were convinced that a blood sugar test was helpful, whereas 43 participants (6.7%) saw the test as unhelpful. In the survey, 492 individuals (76.8%) thought that the cost of a blood sugar test was not expensive. However, 62 (9.7%) did not perform a blood sugar test because it was costly. While 521 (81.3%) did not feel that the blood sugar test was painful, 54 of the participants (8.4%) regarded it as painful. Moreover, 412 respondents (64.3%) said they were not afraid of learning the result of the blood sugar test, while 130 (20.30%) thought that the prospect of learning the result made them nervous.

**Table 5:** Prediabetic screening barriers faced by the participants

Barrier	Disagree n (%)	Neutral n (%)	Agree n (%)
I did not take a blood sugar test because it is not available.	397 (61.9)	123 (19.2)	121 (18.9)
I did not take a blood sugar test due to the lack of time.	340 (53)	122 (19)	179 (27.9)
I did not take a blood sugar test because I am not convinced of its usefulness.	529 (82.5)	69 (10.8)	43 (6.7)
I did not perform a blood sugar test because I think it is expensive.	492 (76.8)	87 (13.6)	62 (9.7)
I did not perform a blood sugar test because I feel that it is painful.	521 (81.3)	66 (10.3)	54 (8.4)
I did not perform a blood sugar test because I am afraid of learning the result.	412 (64.3)	99 (15.4)	130 (20.3)

Sociodemographic characteristics and knowledge of prediabetes

Table 6 presents the relationship between participants' sociodemographic characteristics and their knowledge levels of prediabetes. In terms of sex, most of the male (191, 61.4%) and female (208, 63%) participants possessed sufficient knowledge of prediabetes. This finding suggests that there is no significant difference between the knowledge levels of men and women.

**Table 6:** Association between the participants' sociodemographic characteristics and knowledge of prediabetes

Characteristic	Knowledge level			P-value
	Poor n (%)	Moderate n (%)	Good n (%)	
Sex				
Male	21 (6.8)	99 (31.8)	191 (61.4)	0.524
Female	28 (8.5)	94 (28.5)	208 (63)	
Age				
<25 years	13 (7.5)	67 (38.5)	94 (54)	0.027
25–40 years	21 (10)	55 (26.1)	135 (64)	
>40 years	15 (5.9)	71 (27.7)	170 (66.4)	
Educational level				
High school and below	17 (13.3)	45 (35.2)	66 (51.6)	<0.001
Diploma	11 (15.1)	28 (38.4)	34 (46.6)	
Bachelor's degree	18 (4.6)	108 (27.8)	262 (67.5)	
Master's degree	3 (5.8)	12 (23.1)	37 (71.2)	
Occupation				
Student	10 (7.4)	48 (35.3)	78 (57.4)	0.182
Government official	18 (7.2)	59 (23.5)	174 (69.3)	
Private-sector employee	8 (8)	35 (35)	57 (57)	
Unemployed	5 (12.8)	16 (41)	18 (46.2)	
Retired	2 (4.8)	13 (31)	27 (64.3)	
Homemaker	6 (8.2)	22 (30.1)	45 (61.6)	
Education in the medical field				
Yes	6 (4.7)	29 (22.5)	94 (72.9)	0.019
No	43 (8.4)	164 (32)	305 (59.6)	
Monthly family income				
<3,000 SAR	20 (10.4)	68 (35.4)	104 (54.2)	0.009
3,000–5,999 SAR	4 (4.5)	35 (39.3)	50 (56.2)	
6,000–8,999 SAR	5 (6.9)	24 (33.3)	43 (59.7)	
9,000–12,000 SAR	8 (10.3)	19 (24.4)	51 (65.4)	
>12,000 SAR	12 (5.7)	47 (22.4)	151 (71.9)	

Regarding age, a statistically significant relationship exists between age groups and knowledge levels. Participants over 40 years of age (170, 66.4%) represented the largest proportion of participants with sufficient knowledge, while those below 25 years represented the least proportion of participants (94, 54%) with sufficient knowledge. In terms of educational level, the participants with sufficient knowledge as well as bachelor's degrees and master's degrees amounted to 262 (67.5%) and 37 (71.2%), respectively. This suggests that higher education levels may contribute to a better understanding of prediabetes among the participants.

Moreover, occupation does not show a significant relationship with knowledge. However, it is worth noting that government officials (174, 69.3%) and students (78, 57.4%) represented a larger proportion of participants possessing sufficient knowledge compared to those holding other occupations. Participants with an education in the medical field (94, 72.9%) represented a significantly larger proportion of participants with sufficient knowledge compared to those without such an education (305, 59.6%). 

Finally, monthly family income also shows a statistically significant relationship with knowledge level. As the monthly family income increases the level of good knowledge about prediabetes increases. The participants with a monthly income greater than 12,000 SAR (151, 71.9%) represented the largest proportion of participants with sufficient knowledge, while those with an income below 3,000 SAR (104, 54.2%) represented the smallest proportion of participants with sufficient knowledge.

## Discussion

According to this study's results, the participants' knowledge levels of prediabetes were found to be good (62.2%), moderate (30.1%), and poor (7.6%). The results indicate that there is a solid foundational awareness of prediabetes, which is significantly associated with age, educational level, education in the medical field, and monthly family income. Approximately two-thirds of the participants correctly identified prediabetes as a condition through which blood glucose levels are elevated but are not high enough for a diabetes diagnosis. Similar to a study assessing the prediabetes knowledge of Saudi adults in the Al Ahsa region, this study included a significantly high percentage of respondents (87.1%) with a high level of knowledge about prediabetes [6]. In contrast, a study of people over 45 years of age found that their knowledge scores were good (23.2%), moderate (46.3%), and poor (30.5%) [5]. Additionally, another study found significant gaps in primary care physicians' knowledge of prediabetes in Saudi Arabia, contributing to the condition being insufficiently screened and undertreated [7].

This study's results also suggest that there are gaps in participants' knowledge of the specifics of prediabetes, such as its potential progression to diabetes and the precise blood sugar levels that define the prediabetic stage. For instance, only 39% were aware that it could progress to type 2 diabetes if not managed properly. Moreover, in this study, 40.2% of the participants had prior knowledge of sugar levels in the prediabetic stage. A study conducted in South India found that 25.6% of its participants were aware that prediabetes can progress to type 2 diabetes and that only 29.9% of them answered correctly when asked what the fasting blood sugar level was for the prediabetic stage [8]. This indicates a need for more in-depth education about the long-term risks of prediabetes. Studies conducted in India, the United States, and Bahrain have highlighted the importance of education programs and screening guidelines to improve the knowledge and management of prediabetes [8-10].

In this study, the mean number of respondents who had positive attitudes was 468 (73%), and that of negative attitudes was 173 (27%). Most participants (73.2%) agreed that keeping blood sugar levels close to normal is important, emphasizing that this is crucial for maintaining good health and preventing the progression of type 2 diabetes. A large proportion of them (88.3%) also recognized the necessity of education, highlighting the fact that being well-informed about prediabetes can empower individuals to make better health choices. Additionally, the participants (88.9%) acknowledged the significant role of family support, noting that encouragement and assistance from family members can make the management of prediabetes easier. They (65.8%) believed in the possibility of leading a normal life despite having prediabetes, expressing optimism about continuing their daily routines and activities without major disruptions. However, Many of them (57.6%) believed that controlling blood sugar levels was not particularly challenging in the case of prediabetes. Furthermore, there is a perception among 61.5% of the participants who did not believe that, even if the blood sugar level is successfully controlled during the prediabetes stage, progression to T2DM is inevitable. Further, a significant majority of the participants (76%) felt that the condition of prediabetes is largely ignored by society. They believed that there is a lack of awareness and concern about prediabetes, which leads to insufficient support and resources for individuals dealing with this condition. In contrast, according to Borba et al., more than 85% of Brazilian seniors maintain negative attitudes toward diabetes [11]. Additionally, a study found that 50% of senior individuals showed negative attitudes toward lifestyle changes to improve prediabetes [12].

Based on the mean values, it is evident that the current practices related to diet and lifestyle among the participants are generally poor 538 (84%), and only 103 (16%) had good practice. This study revealed that only (32.9%) of the respondents exercised for three to six hours per week, which is relatively poor. In contrast with the South Indian study, which found that just (3.9%) of the respondents exercised for three to six hours per week, after following an educational program, it found that most respondents (62%) exercised for three to six hours per week [8]. However, it also found that 11.7% of the participants followed the poor practice of testing their blood sugar level once every week or once a month, highlighting a potential shortfall in preventive health practices. This finding is similar to the study among people older than 45 years, which found that 18.8% of its participants tested their blood sugar levels at home once every week or once a month [5]. The implication here is that, while there may be general awareness about prediabetes and its risks, there is a lack of practical application of this knowledge in terms of regular blood sugar monitoring. 

Moreover, in this study, the majority of the participants reported engaging in unhealthy eating behaviors. Only 10.6% of them did not skip meals, while just 8.3% did not consume excessive fat. Furthermore, 14.5% of the participants had substituted fiber-rich foods, and 19.2% had not added sugar-containing foods to their diets. Further, in this study, only 15.8% of the participants said that they never slept less than six hours a day. However, it was shown that 84.2% of them slept less than six hours a night at least one to two times per week, which indicated they had poor sleep habits and experienced poor sleep quality.

A systematic review showed that, while most healthcare providers educate patients on lifestyle changes, the content of their advice could vary. Some (67.7-97.9%) only talked about dietary management, while others (21.8-98.6%) offered advice on diet and exercise [13].

Based on the mean number of respondents, this study found that most participants 449 (70%) do not believe any barrier exists to prediabetic screening. However, some participants (18.9%) did not perform a blood sugar test because it was not available, while others (27.9%) did not perform a blood sugar test due to a lack of time. Some participants (20.3%) did not perform a blood sugar test because they were afraid of learning the results of the early test. These findings emphasize the need for more convenient and accessible prediabetic screening services, as well as for public education efforts to highlight the importance of making time for health screenings.

Limitations

Despite the valuable insights derived from this study, it is crucial to recognize its limitations. First, the study utilized a self-administered questionnaire for data collection, which may have introduced recall bias. Participants might have inaccurately remembered or reported their practices and attitudes, potentially affecting the reliability of the information gathered. Second, the study has chosen to concentrate solely on Saudi citizens as its subject of research, which consequently limits how broadly its findings can be applied to other populations in the region. Finally, the use of a convenience sampling method could result in a sample that does not accurately reflect the wider population, possibly affecting how well the study represents the overall group.

## Conclusions

This research provides valuable insights into the demographic characteristics, knowledge, attitudes, practices, and barriers related to prediabetes among the surveyed participants. The findings underscore the importance of developing targeted interventions and strategies to improve the knowledge and awareness of prediabetes, promote healthier lifestyle practices, and address barriers to the screening of prediabetes. By addressing these factors, healthcare professionals and policymakers can work toward preventing the progression of prediabetes to type 2 diabetes and ultimately improving overall public health outcomes. It is crucial to implement evidence-based interventions that consider the specific needs of different demographic groups, ensuring equitable access to education, screening, and support services. This study particularly highlights the importance of health education initiatives for enhancing understanding and attitudes toward prediabetes among the Saudi population.

Given this study's findings about barriers to prediabetes screening, future research should focus on uncovering effective strategies for overcoming the identified barriers to prediabetes screening. These barriers include perceived complexity in managing prediabetes, apprehension of diagnosis, and a lack of time for health check-ups. This study's findings have profound implications for health policies and diabetes prevention efforts within Saudi Arabia, providing a basis for subsequent research and targeted interventions.
